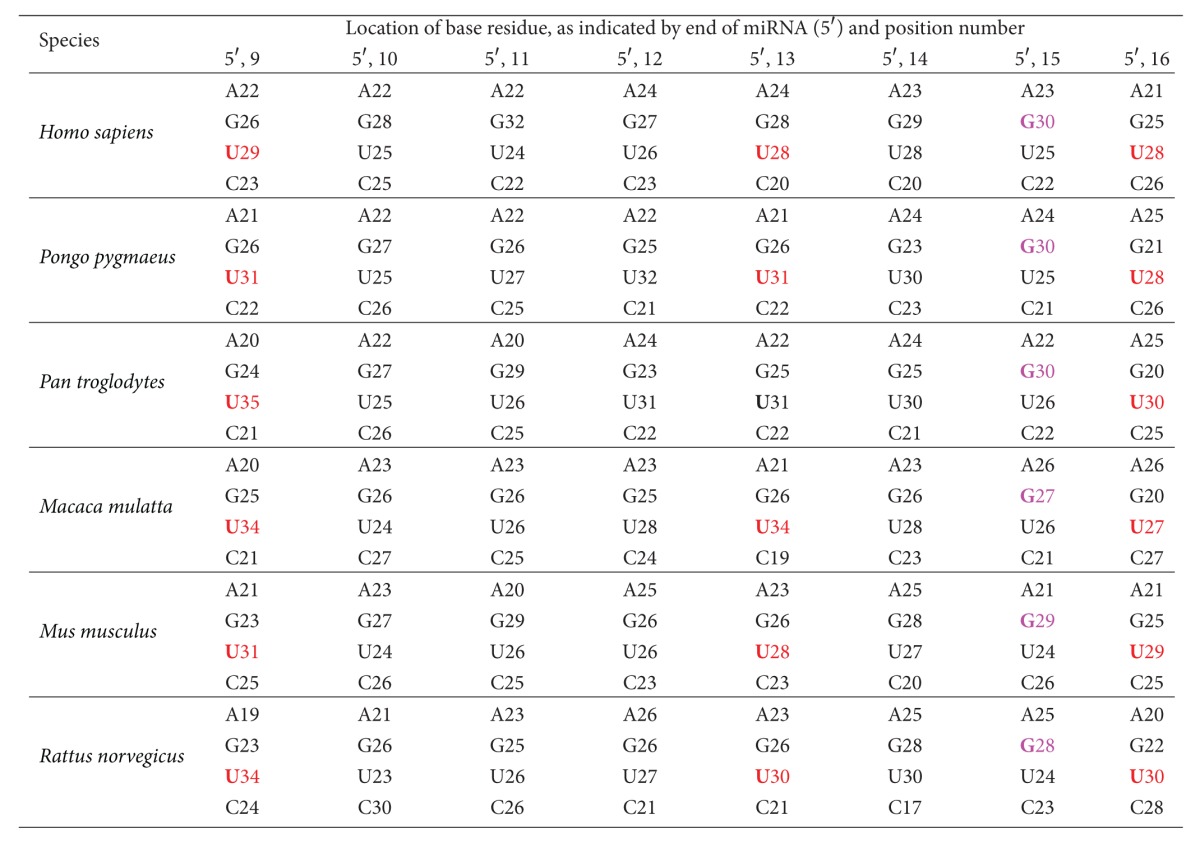# Base Composition Characteristics of Mammalian miRNAs

**DOI:** 10.1155/2013/951570

**Published:** 2013-02-24

**Authors:** Bin Wang

**Affiliations:** Department of Chemistry, Robert C. Byrd Biotechnology Science Center, Marshall University, Huntington, WV 25755, USA

## Abstract

MicroRNAs (miRNAs) are short RNA sequences that repress protein synthesis by either inhibiting the translation of messenger RNA (mRNA) or increasing mRNA degradation. Endogenous miRNAs have been found in various organisms, including animals, plants, and viruses. Mammalian miRNAs are evolutionarily conserved, are scattered throughout chromosomes, and play an important role in the immune response and the onset of cancer. For this study, the author explored the base composition characteristics of miRNA genes from the six mammalian species that contain the largest number of known miRNAs. It was found that mammalian miRNAs are evolutionarily conserved and GU-rich. Interestingly, in the miRNA sequences investigated, A residues are clearly the most frequent occupants of positions 2 and 3 of the 5′ end of miRNAs. Unlike G and U residues that may pair with C/U and A/G, respectively, A residues can only pair with U residues of target mRNAs, which may augment the recognition specificity of the 5′ seed region.

## 1. Introduction

MicroRNAs (miRNAs) are single-stranded, short (15–27 nucleotides) RNA sequences that repress protein synthesis via base pairing to some portion of a messenger RNA (mRNA), such as the 3′ untranslated region (3′UTR), the 5′ untranslated region (5′UTR), or the coding region [1, 2]. Endogenous miRNAs have been found in various organisms, including animals, plants, and viruses. In mammals, precursor miRNAs (pre-miRNAs) 60–100 nucleotides in length form a hairpin stem-loop structure and are processed by Dicer, a highly conserved RNase III family endonuclease that is found in almost all eukaryotic organisms [3, 4]. This processing yields a miRNA : miRNA duplex that is approximately 21 nucleotides long. One or both of the duplex's short RNA strands is incorporated into RNA-induced silencing complexes (RISCs, which are composed of Dicer, Argonaute, and other nonspecified proteins) and functions as a mature miRNA that can base pair with mRNA targets, inducing either the degradation or translational repression of the mRNA [3, 4].

The regulation of miRNA is essential for organisms because miRNA plays critical roles in numerous biological processes, including the proliferation and differentiation of cells, as well as apoptosis. It is predicted that mammalian miRNAs can regulate approximately 30% of protein-coding genes [4]. The dysfunctional posttranscriptional modulation of gene expression caused by miRNAs has been related to diseases such as cancer and neurodegenerative disorders [5–8]. The investigation of miRNAs and their gene targets has thus attracted much attention in recent years and has become a major focus of research in medicine and molecular biology [7, 9–13].

For this study, the author explored the base composition of mature miRNAs from the six mammalian species that contain the largest number of known miRNAs. Position-specific base dominance in certain miRNAs has been investigated by other researchers; for example, Gkirtzou et al. discussed the sequence composition on and around mature miRNAs for humans and mice [14], and Wang et al. described position-specific base dominance in human miRNAs [15]. However, this paper provides more complete, detailed, and updated information regarding the base composition characteristics of mature miRNAs in four primates and two rodents, thus providing additional knowledge regarding mammalian miRNAs. We found that the mammalian miRNAs are evolutionarily conserved and GU-rich, where U residues are more frequent than other bases. Adenosine residues are generally a less frequent occupant, except at positions 2 and 3 in the 5′ seed region of these miRNAs. This characteristic may explain the specificity of pairing and target recognition by the 5′ seed region in miRNAs.

## 2. Materials and Methods

The sequences of mature miRNAs were obtained from miRBase (http://www.mirbase.org/), a database that contains experimentally supported base sequences for various miRNAs [16–19]. The miRNA data for *Homo sapiens*, *Pongo pygmaeus*, *Pan troglodytes*, *Macaca mulatta*, *Mus musculus*, and *Rattus norvegicus* were exported from the miRBase database and then transferred into Microsoft Excel. In Excel, the miRNA sequences were arranged such that each Excel column contained only the nucleotides at a specific position of the miRNAs studied (e.g., column B contained nucleotides at position 1 of the 5′ end of all human miRNAs; column C contained nucleotides at position 2 of the 5′ end of all human miRNAs studied). The nucleotides in each column were copied and pasted into an online RNA base composition calculator (http://www.currentprotocols.com/WileyCDA/CurPro3Tool/toolId-7.html/) in order to determine the number and percentage of A, G, U, and C at every position of each miRNA of each species studied.

## 3. Results and Discussion

The six largest miRNA pools were exported from the miRBase database, including *Homo sapiens* (1921 miRNAs), *Mus musculus* (1157 miRNAs), *Rattus norvegicus *(680 miRNAs), *Pongo pygmaeus* (600 miRNAs), *Pan troglodytes *(525 miRNAs), and *Macaca mulatta* (488 miRNAs). A small number of human miRNAs were found to have slightly different names but identical sequences. The names of miRNAs in miRBase are first assigned to their hairpin precursors and then inherited by the mature miRNAs. The naming problem is caused by the occasional existence of multiple miRNA members in the same family, where the mature sequence from one stem of the hairpin precursor is slightly different from the same sequence in a miRNA family member, while the sequences from the other stem of the hairpin precursor are identical. After deleting these redundant sequences, 1897 out of 1921 of human miRNAs were included for investigation in this study. No identical sequences were observed within any of the other five species. 

Column 3 in Table 1 provides the total base composition, including total numbers and percentages of A, G, U, and C in all mature miRNAs of each species studied. Overall, G and U residues are more abundant than A and C residues in the miRNA sequences investigated. More Us than Gs are present in five of the six species, with human miRNAs being the exception. Based on canonical Watson-Crick base pairing, this bias in miRNA composition indicates the abundance of A residues in the target mRNA regions.

David Bartel's research team discovered the high frequency of U and low occurrence of G at the 5′ end of *Caenorhabditis elegans* miRNAs [20]. Data shown in Table 1, column 4, confirmed this tendency in mammalian species. At position 1 of all experimentally confirmed miRNAs in each species studied, the frequency of U residues ranges from 36% to 44% (shown in red in Table 1), whereas G residues range from 11% to 14% (shown in green in Table 1). In addition, C residues are less frequent at the 5′ end of mammalian miRNAs, indicating a preference for base pairs with lower thermodynamic stability (i.e., favoring AU rather than GC base pairs) at position 1.

Data presented in Table 1, column 5, provide the base composition at the 3′ end of the miRNAs studied. Interestingly, the 3′ end demonstrates the same abundance of U residues as found in the 5′ end of miRNAs, indicating an enrichment of A residues within the 5′ end of mRNA targets. G and C residues are less frequent in the 3′ end of miRNAs as well, however, to a lesser degree than in the 5′ end.

It has been reported that the presence of an A residue in the mRNA target, across from position 1 of the 5′ end of a miRNA, improves binding between the miRNA and its target. This occurs even though actual base pairing between the A residue and the nucleotide at position 1 of the 5′ end of the miRNA is not necessary [3, 4, 21–24]. Fewer studies have focused on the 3′ end of miRNAs [14]. The strong bias toward U residues at position 1 of both ends of miRNAs in the six mammalian species studied in this paper implies that both ends, rather than just the 5′ end, may participate in target recognition. One study demonstrated that miRNA can simultaneously interact with both the 3′UTR and 5′UTR of a mRNA target via complementary base pairing using its 5′ and 3′ regions, respectively [25], thus providing evidence for the possibility that both ends of miRNAs are involved in target recognition.

In metazoans, the second-through-seventh nucleotides at 5′ end of a miRNA are known as the seed region, which is the most important region for miRNA complementarity and target recognition [23, 26]. In this study, we investigated the base composition (i.e., the percentages of A, G, U, and C) for two areas of every mature miRNA of each species in our study. We examined the sequences both the 5′ seed region and the nucleotide immediately following it (i.e., the eighth nucleotide at the 5′ end); our results are presented in Table 2. The corresponding regions at the 3′ end were also studied, and those results are also presented in Table 2. In the 5′ seed region, there is a preference for A at positions 2 and 3 (shown in red in Table 2), followed by a preference for G at positions 4–6 (shown in magenta in Table 2). The base preference at positions 7 and 8 is not consistent across the six species. In the corresponding 3′ regions, there is a strong preference for G at position 2 (shown in orange in Table 2), followed by a preference for U at position 3 (shown in green in Table 2). Position 4 at the 3′ end of these miRNAs is more frequently G in primates and U in rodents (shown in blue in Table 2). Position 5 at the 3′ end of these miRNAs is most frequently U (shown in purple in Table 2); U is also the most frequent occupant of position 7 (shown in purple in Table 2), except in human miRNAs. The base preferences at positions 6 and 8 of the 3′ end of miRNAs are not consistent across the six species studied.

The specificity of miRNA targeting in mammals is not necessarily restricted to the 5′ and 3′ ends of the seed pairing regions, and previous reports suggest that seed matches are not always sufficient for the repression of targets in mammals [3]. Therefore, base composition beyond the eighth nucleotide from the 5′ end of miRNAs was also investigated in this study. Base composition information for the ninth-through-sixteenth nucleotides from the 5′ end of the miRNAs is presented in Table 3. Positions 9, 13, and 16 demonstrate a strong preference for U; whereas position 15 shows a strong preference for G in all six species investigated. The base preferences at positions 10, 11, 12, and 14 from the 5′ end of the miRNAs are not consistent in these six species.

Previous studies suggest that the presence of an A or U residue in the mRNA target, across from position 9 of the 5′ end of a miRNA, improves binding between the miRNA and its target. This occurs even though the A or U does not need to base pair with the nucleotide at position 9 of the 5′ end of the miRNA [3, 4, 22]. Our study demonstrates that at position 9 of the miRNAs, there is a strong preference for U (ranging from 29% to 35% in the six species investigated), which is able to base pair with the A residue in the mRNA target. This finding implies that the nucleotide at position 9 of the 5′ end of a miRNA plays an important role in improving both the recognition of specific targets and the functional efficiency of the miRNA.

It has been reported that residues at positions 13–16 of the 5′ end of the miRNAs are also important for stabilizing miRNA-mRNA interactions, especially when base pairing in the 5′ seed region is suboptimal [3, 4, 22]. The lengths of the mature miRNAs investigated in this study range from 15 to 27 nucleotides. For some short miRNAs, residues at positions 13–16 are located in the 3′ half of the miRNAs; however, for some long miRNAs, these residues are situated in the central region of the miRNAs. Therefore, whether nucleotide pairing at positions 13–16 of the 5′ end of a miRNA is important may, at least partially, depend on their exact locations in the miRNA investigated.

Overall, mammalian miRNAs are evolutionarily conserved and GU-rich, where U residues are more frequent than other bases. This finding implies that their target mRNA sites should be A-rich. Another interesting finding is that, in the miRNA sequences investigated, A residues are clearly the most frequent occupants of positions 2 and 3 of the 5′ end of miRNAs. Unlike G and U residues that may pair with C/U and A/G, respectively, A residues can only pair with U residues of target mRNAs, which may augment the recognition specificity of the 5′ seed region.

## Figures and Tables

**Table 1:** Total base composition and base composition at position 1 of both ends of mature miRNAs in six mammalian species. Bases shown in red represent the most frequent residues in position 1 of both ends of the miRNAs, whereas bases in green indicate the least frequent residues in position 1 at the 5′ end of these miRNAs.

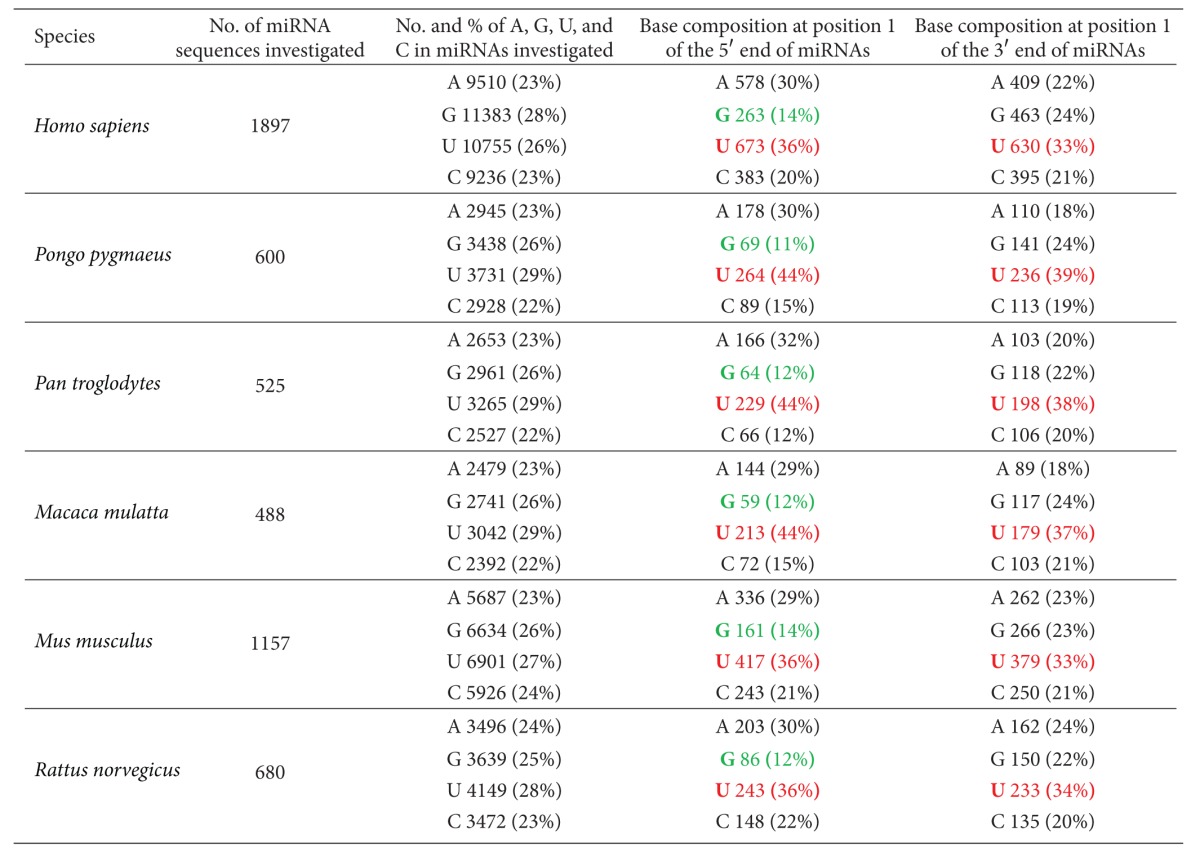

**Table 2:** Percentages (%) of A, G, U, and C in every mature miRNA of each species studied. This table contains base composition information for the second-through-eighth nucleotides from both ends of the miRNAs. Bases shown in red are the most favored residues at positions 2 and 3 of the 5′ end; bases in magenta are the most favored residues at positions 4–6 of the 5′ end; bases in orange are the most favored residues at position 2 of the 3′ end; bases in green are the most favored residues at position 3 of the 3′ end; bases in blue are the most favored residues at position 4 of the 3′ end; and bases in purple are the most favored residues at positions 5 and 7 of the 3′ end of the miRNAs examined.

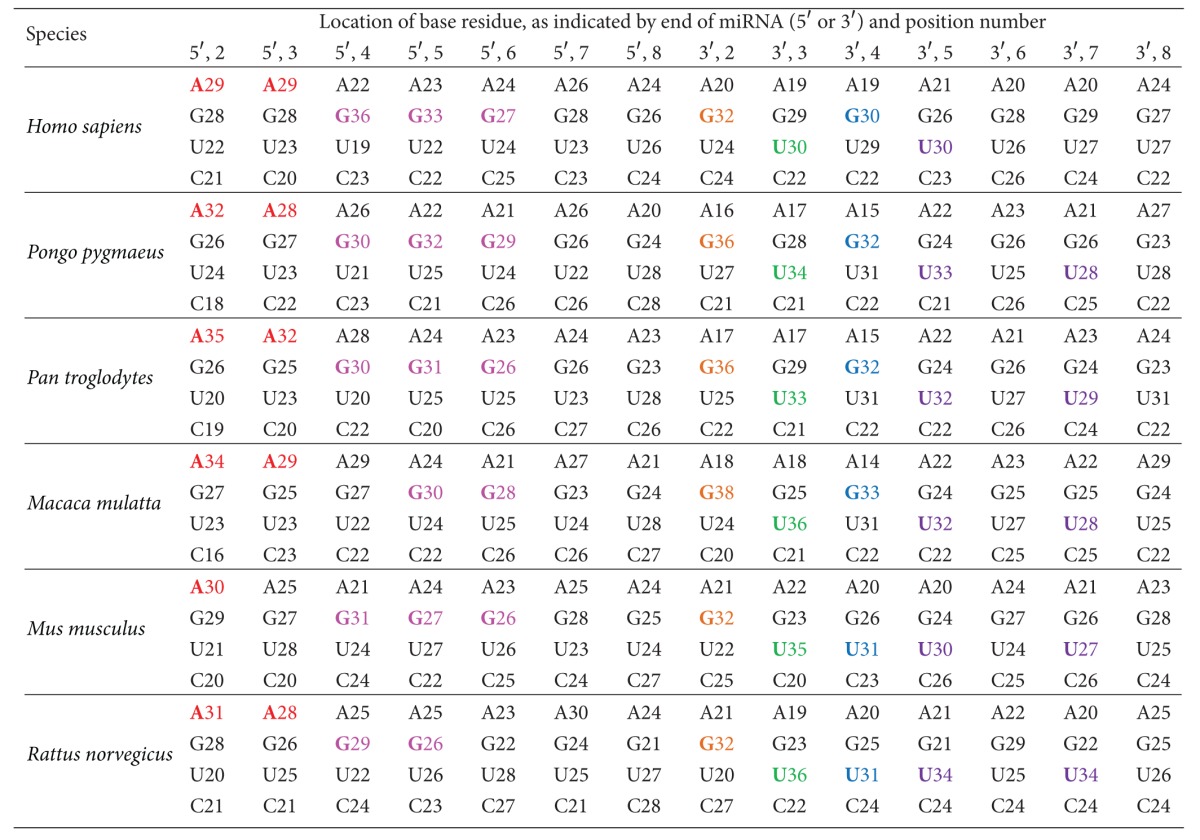

**Table 3:** Percentages (%) of A, G, U, and C in every mature miRNA of each species studied. This table contains base composition information for the ninth-through-sixteenth nucleotides from the 5′ end of the miRNAs. Bases shown in red are the most favored residues at positions 9, 13, and 16 of the 5′ end; bases in magenta are the most favored residues at position 15 of the 5′ end of the miRNAs examined.